# Enhancing celery’s growth, production, quality, and nutritional status using tryptophan and glycine amino acids

**DOI:** 10.1038/s41598-024-76421-x

**Published:** 2024-11-04

**Authors:** A. M. M. El-Tanahy, Sami H. Mahmoud, Mohamed S. A. Abd Elwahed, Dina M. Salama

**Affiliations:** 1 Vegetable Research Department, Agricultural and Biology Research Institute, National Research Centre (NRC), 33 El-Buhouth St., Dokki, Giza, 12622 Egypt; 2 Botany Department, Agricultural and Biology Research Institute, National Research Centre (NRC), 33 El-Buhouth St., Dokki, Giza, 12622 Egypt

**Keywords:** Celery, Amino acids, Tryptophan, Glycine, Yield, Quality, Nutritional status, Plant sciences, Plant development, Plant physiology

## Abstract

Finding a way to establish a sustainable cultivation system to produce celery as an important source of human being nutrition system due to its health and nutritional advantages is increasing by the day. Amino acids have a deep impact on plant metabolism; they improve mineral uptake and increase shoots and root growth, yield, chlorophyll biosynthesis, and photosynthesis rate as well as encouraging stimulation of several enzymes and coenzymes which lead to improved plant development and production besides quality. A two-year (2021/2022–2022/2023), trial was conducted to discuss two essential amino acids Glycine (GLY) and Tryptophan (TRP) effect on celery’s growth, production, photosynthetic pigments, vitamin (C), total phenols, total flavonoids, total antioxidant activity, total indoles, nutritional status, and amino acids contents. During the winter season, three levels of each amino acid (0, 50, and 75 mg/l) were sprayed in two doses after 30 days of cultivation and 15 days from the first foliar. Results showed that the best performance of amino acids on plants vegetative growth characteristics, photosynthetic pigments, biochemical constituents, yield, and amino acids content was conducted by using (GLY + TRP) mix followed by TRP then GLY, while the best level of foliar applied amino acids was 75 mg/l in concentration. The best results on celery’s vegetative growth, yield, chemical content, and amino acid content were recorded by using the (GLY + TRP) mix at the highest level of 75 mg/l in concentration.

## Introduction

Celery (*Apium graveolens* L.) is a member of the family “Apiaceae”, it has been growing in the Mediterranean area and southern part of Europe for thousands of years as this part of the world is considered the origin place of celery^1,2^. In Egypt, the total cultivated area of celery in the winter season is 36 hectares with a total production of 828 tons (22.9 ton/hectare). Nowadays, the demand for celery in Egypt has increased due to its importance for exportation as well as covering the internal market especially that part serving tourists visiting the country. Celery stalks and leaves can be consumed fresh in salad, dry, or cooked as soup and its seed has been used in traditional medicine^3,4^. It has medicinal benefits such as using against asthma bronchitis and swollen glands. Also, it served as a laxative or sedative besides being used as a stimulant, and tonic^5,6^. Celery contains several healthy components such as fibers, vitamins, minerals, and amino acids which are very important and beneficial for human health^1,6^. Its leaves contain several vitamins such as C, K, and A, besides carotenoid components especially flavonoids like apigenin and luteolin, that have anticancer effects^7^.

Amino acids are recognized as a major group of active substances that can be used as biostimulants, where they are easy to absorb and affect plant growth^8^.Theystimulate plants’ natural nutrition processes^9^. They act as signals for many metabolic processes encouraging a better plant to assimilation of nitrogen^10^. They are the peptide components’ building blocks, serving as protein synthesis substrate^11^, improving minerals uptake from either foliar or soil-added fertilizers^12^, increasing shoots and roots growth, yield, chlorophyll biosynthesis and photosynthesis rate^13^, and encouraging stimulation of several enzymes and coenzymes^14^.

Tryptophan (TRP) is an amino acid that plays a unique role in building IAA rings^9^. It might be added to soil, foliar spray, or seed priming^15^. It has a positive effect on plant growth by controlling IAA release^16^. Its remarkable effect on auxin synthesis was recorded after either foliar or soil application^17^. Yasmin et al.^18^ using TRPincreases many hormone levels in maize cells, i.e., IAA, GA, and ABA either in normal or under stress conditions.El-Kenawy^19^noticed an increase in vegetative growth and yield after spraying TRP. Its foliar application led to a rise in both total chlorophyll and carotenoid contents and improved vegetative growth and chemical content of Pelargonium graveolens, Wheat, Lupine, and Moringa seedlings^20^.Rahman et al.^21^ said that TRP increases ascorbic acid inpepper fruits and lettuce leaves. Foliar TRP application increased protein and total sugar content in Brassica napus as mentioned by Dawood and Sadak^22^.

Glycine (GLY) is an amino acid that plays a valuable role in promoting photosynthesis and its efficiency by enhancing chlorophyll formation and encouraging vegetative growth besides its function in pollination and fruitfulness^23^.By far, it is considered the highest widely used amino acid in plant nutrition^24^. Several factors control itsapplication effectiveness including plant species, growth stages, climatic conditions, and both application number and concentration^25^. Also, amino acids, including GLY, have a valuable impact on both the yield and quality of leafy plants^26,27^**.** Shooshtari et al.^24^ and Souri & Hatamian^25^ mentioned that the application of amino acids like GLY introduces more sustainable and safer production compared with chemical fertilizations;at present time, it has been playing a very valuable role in several plants’ nutritional management, in particular for horticultural crops.The GLYtreatments improved growth besidesthe chemical composition of coriander in several plant species^28^, as in lettuce^27^, sweet basil^29^,and celery plants^30^.

Nowadays, vegetable producers are aiming to increase vegetable productivity and quality to cover the increasing demand by using sustainable agriculture practices and enhancing crop productivity^31^. Thus, the objectives of the research are to use the amino acid GLYbecause of its important role in the process of photosynthesis, as it is involved in the composition of the chlorophyll molecule, and the amino acid of TRP is used, which builds important plant hormones for the plant, such as the auxin hormone that stimulates plant growth. Consequently,our study aims to use amino acids (GLYandTRP)to promote celery’s chlorophyll content to encourage growth and production as well as enhance its quality and nutritional status to establish a sustainable pathway for celery’s production. Finally, we can decidethat the targetof our study is new because amino acids (GLY and TRP) have not been studied as a foliar application on French celeryplant, and its content of phenolics, flavonoids, indoles, antioxidants, amino acids, and nutrients.

## Materials and methods

### Experiment site

**Fig. 1:**
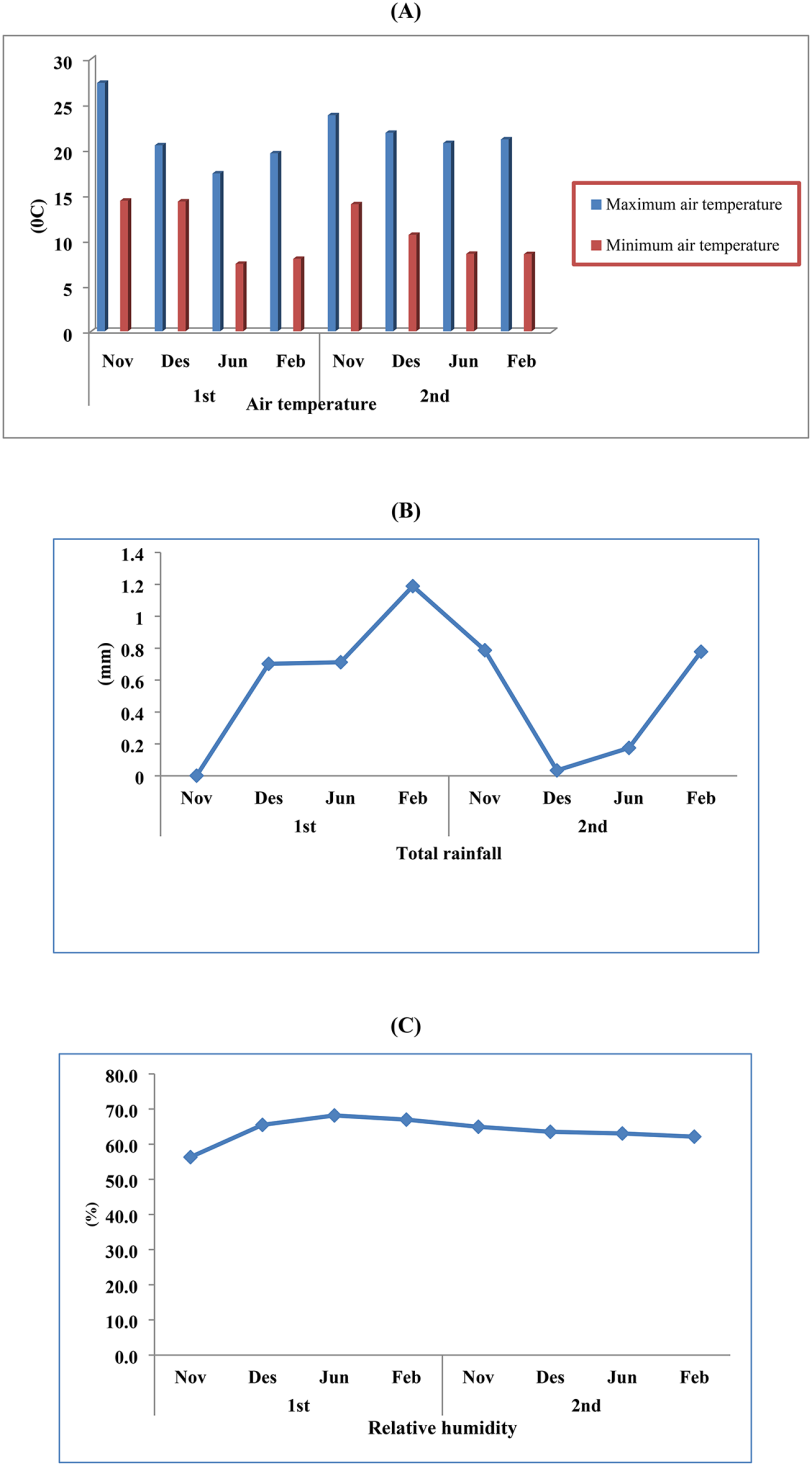
Metrological data (monthly minimum and maximum (**A**) air temperature, total rainfall (**B**), and relative humidity (**C**) during two experimental seasons 2021/2022–2022/2023.

Field trial was conducted during the two winter seasons of 2021/2022 and 2022/2023 in a new reclaimed sand soil field at NRC Experimental ranch in El-Nubaria, El-Behira Governorate, Egypt (30°29′50"N 30°19′16"E) aiming to investigate the effect foliar applications of amino acids (GLY and TRP) regarding growth, yield, biochemical constituents as expressed as vitamin (C), total phenols, total flavonoids, total antioxidant activity, total indoles, and nutritional status, and amino acids contents of celery plants. Both physical and chemical analyses of trail soil are shown in Table1. The investigational soil was analyzed in agreement with the methods illustrated by Cottenie et al.^32^. Metrological data as expressed as minimum and maximum air temperature, total rainfall, and relative humidity were calculated with monthly averages during two experimental seasons 2021/2022–2022/2023 are shown in Fig. 1**.** Metrological data were obtained from the Central Laboratory for Agriculture Climate.

**Table 1:** Physical and chemical properties of experimental soil (Combined data of two seasons).

Soil physical properties								
Texture	(%)							
	Sand	Silt	Clay					
Sandy	92.1	0.83	7.07					
Soil chemical properties								
EC (ds/m)	pH	OM (%)	(meq/1)					
			Ca	Mg	Na	K	CL	HCO_3_
1.8	8.2	0.44	7.06	0.54	0.99	0.32	0.52	1.31

### Experiment treatments

Seedlings of celery cv. Fransawi were transplanted on the third of November in each growing season. All the plant material that was used as samples in our present experiment was collected from plants cultivated, grown, and then harvested at NRC Experimental Ranch in El-Nubaria, El-Behira Governorate, Egypt. The NRC approved the experiment to take place on its farm and gave its permission for all processes and for collecting plant samples as well. The whole experiment including collecting plant samples was conducted under the supervision and with the permission of the vegetable research department, agriculture, and biological institute, NRC in Cairo, Egypt, and according to its ‘Ethics approval and consent to participate’ section. The source of the celery plants used in this experiment is imported seeds from overseas which are marketed and sold commercially in the Egyptian market as Celery cv. Fransawi that cultivar was the subject of this investigation in our experiment. These plants are not wild and according to Egyptian law, we do not have to deposit them in a public herbarium. Also, the study on these plants was based on evaluating the vegetative growth, and no work was done in the study on seeds. Nevertheless, the rest of the dry samples of the vegetative parts which were used in the chemical and physical analysis were saved in the storage section of the vegetable research department, agriculture, and biological institute, NRC in Cairo, Egypt and that is either for 3 years period or until the date gets published.

Three levels of both amino acids (GLY and TRP) using concentrations of 0, 50, and 75 mg/l were applied as a foliar application of two doses after a month of sowing seedlings cultivation and 15 days from the first foliar. Drip irrigation laterals used for seedlings planting as 1 m apart, 25 m long, and 30 cm between drippers (standard 4 L/h discharge @ 1.5 bar drippers), seedlings were planted around each dripper with10 cm apart. seedlings. Celery seedlings were cultivated on both sides of the line.

Regarding fertilizing celery plants, celery plants were fertilized with 238 kg of ammonium sulphate + 238 kg of super phosphate + 119 kg of potassium sulphate. This is in addition to organic fertilizer in the amount of 47.6 cubic meters per hectare. The chemical fertilizer was applied in two batches, the first three weeks after planting and the second two weeks after the first, while the organic fertilizer was added during the land service. The fertilization, irrigation, and celery plant resistance to weeds and diseases were carried out as the Egyptian Ministry of Agriculture recommended.

### Experimental design

The experiment consisted of 9 treatments consisting of 3 concentrations of GLYand 3 concentrations of TRP with three replicates. The treatments were randomly arranged in a split-plot design system, as GLY levels took place in the main plots while TRP levels were in the subplots.

### Measurements of crop parameters

Data Collection: At harvest time on February 16, a random sample of 5 plants from each replicate of all treatments was collected and then transferred to the laboratory of the Vegetable Research Department at the NRC for determination of the following traits:

#### Plant vegetative growth characteristics

Plant length (cm), leaves number per plant, plant fresh weight (g/plant), and dry matter (%).

#### Yield

Yield (ton/hectare).

#### Photosynthetic pigments

(Chlorophyll a, b, total chlorophyll, and carotenoids) in fresh leaf tissue of celery plants were calorimetrically determined at 70 days after sowing. Chlorophyll a, b, total chlorophyll, and carotenoid contents in apical head tissues were estimated in representative fresh leaf samples. The obtained extracts were measured spectrophotometrically at wavelengths of 663, 647, and 470 nm, using N, N- Dimethylformamide as a blank according to Moran^33^.

#### Biochemical constituents

##### Vitamin (C)

Content was measured in the fresh weight of the celery plant. Plant samples were extracted using 1% oxalic acid. The extracts were titrated against a solution containing 295 mg/l DPIP (2,6-dichlorophenolindophenol) and 100 mg/l sodium bicarbonate according to the method mentioned in AOAC^34^.

##### The phenolics content

The content of phenolics in celery plants (leaves and stalk) was evaluated on a dry weight basis using a spectrophotometric method. Phenolics were extracted by ethanol 80%. Phenolics were determined by adding 1 ml of sample, 70 ml distilled water followed by Folin–Ciocalteau reagent, and 15 ml of saturated sodium carbonate solution, incubated at room temperature for 30 min and measured at 765 nm in a spectrophotometer. Gallic acid was used to make the calibration curve according to Stratil et al.^35^.

##### Total flavonoids

The content of flavonoids in dried celery plant was estimated by adding 0.5 ml of sample (Flavonoids were extracted by ethanol 80%), 10% aluminum chloride (0.1 ml), 1 M potassium acetate (0.1 ml), and distilled water (4.3 ml) were mixed. After incubation at room temperature for 30 min, the absorbance was measured at 415 nm using a spectrophotometer^36^. Quercetin was used to make the calibration curve.

##### Total antioxidant activity

It was determined in leaves. A stock solution was prepared by dissolving 24 mg 1,1-diphenyl-2-picrylhydrazyl (DPPH) with 100 ml methanol and then stored at 20°C until needed. The solution was obtained by mixing 10 ml stock solution with 45 ml methanol to absorb 1.1 ± 0.02 units at 515 nm. Extracts (750 μl) were allowed to react with 1,500 μl of the DPPH solution for 5 min in the dark. Then, the absorbance was taken at 515 nm. The standard curve was linear between 25 and 800 μmol Trolox^37^.

##### Total indoles

Estimated by adding 1 ml of ethanol extract sample into a test tube followed by 2 ml of Salkowski reagent (consisting of 150 ml concentrated H_2_SO_4_ and 7.5 ml FeCl_3_.6H_2_O 0.5M) was subjoined^38^. This solution was incubated for 30 min at a dark room temperature. Next, total indoles were measured at a wavelength of 530 nm using a spectrophotometer^39^. The concentration of total indoles was determined by using a standard curve using indole-3-acetic acid (IAA).

##### Minerals determination

According to the method mentioned in the AOAC^34^, nitrogen, potassium, and phosphorus contents were estimated using dry celery plant sampleswhich oven dried to a constant weight then it was ground to a fine powder and digested using sulfuric acid and hydrogen peroxide. The concentration of potassium was determined in a digested solution by using flame while the content of phosphorus was evaluated by spectrophotometer, and the percentage of nitrogen was determined by using the Kjeldahl method. As for protein estimation, it is calculated using this equation = N% × 6.15.

##### Amino acids determination

Extraction procedures: 1g sample of the celery dry weight was mixed with 5 mL H_2_O and 5 mL of HCl (final concentration of HCl is 6 M), then was heated at 100°C for 24 h, then was filtered. Lastly, 1 mL of the filtrate was injected into HPLC according to the system as described by Campanella et al.^40^.

HPLC conditions: HPLC analysis was carried out using an Agilent 1260 series. The separation was carried out using Eclipse Plus C18 column (3.0 mm × 150 mm i.d.,3.5 μm). The mobile phase consisted of buffer (sodium phosphate dibasic and sodium borate), pH 8.2 (A), and ACN:MeOH:H_2_O 45:45:10 (B) at a flow rateof 0.64 ml/min. The mobile phase was programmed consecutively in a linear gradient as follows in Table 2:

- The DAD was monitored at 338nm (Bandwidth 10 nm).
- The Fluoresencedetercot was adjusted as the following 340/450 nm (Excitation/Emission)

**Table 2:** Timetable.

Time (min)	A %	B %
0	98	2
0.5	98	2
20	43	57
20.1	0	100
23.5	0	100
23.6	98	2
25	98	2

### Statistical analysis

Data were analyzed statistically, and means were separated using the LSD test at 5% according to the method described by Gomez and Gomez^41^.

## Results

### Plant vegetative growth characteristics

It can be observed from data shown in (Table 3) that amino acid application had positively affected celery’s plant vegetative growth characteristics i.e., plant length, leaves number per plant, plant fresh weight g per plant, and dry matter percentage. The amino acids GLY by using 75 mg/l level recorded the highest values of the previously tested characteristics, those values were significantly higher than other concentrations in both seasons of the trial.

**Table 3:** Effect of GLY and TRP and its different levels on celery’s vegetative growth characteristics during the two seasons of (2021/2022) and (2022/2023).

GLY (mg/l)	TRP (mg/l)	Plant length (cm)		No. leaves/ plant		Plant fresh weight (g/plant)		Dry matter (%)	
		2021/2022	2022/2023	2021/2022	2022/2023	2021/2022	2022/2023	2021/2022	2022/2023
0	0	23.1 ± 0.75	21.1 ± 2.23	16.0 ± 1.00	15.0 ± 0.21	123.5 ± 1.85	117.6 ± 1.55	11.0 ± 0.50	10.6 ± 0.56
	50	25.1 ± 1.21	23.8 ± 1.15	17.0 ± 1.00	16.2 ± 0.95	128.1 ± 2.34	122.4 ± 1.21	12.0 ± 0.15	11.1 ± 0.49
	75	28.3 ± 1.53	26.9 ± 1.45	18.4 ± 0.53	18.0 ± 0.03	133.2 ± 2.52	128.6 ± 1.74	12.5 ± 0.15	11.8 ± 0.15
Mean		25.5	24.3	17.1	16.4	128.3	122.9	11.8	11.2
50	0	25.6 ± 0.75	24.3 ± 0.72	16.0 ± 1.00	15.0 ± 0.65	127.7 ± 1.80	120.7 ± 1.62	12.5 ± 0.42	11.7 ± 0.62
	50	28.8 ± 1.41	27.4 ± 1.34	18.0 ± 1.00	17.2 ± 0.96	140.4 ± 4.93	131.8 ± 2.64	14.2 ± 0.35	13.5 ± 0.40
	75	34.0 ± 1.36	32.3 ± 1.29	21.0 ± 1.00	20.6 ± 0.55	146.0 ± 4.04	138.7 ± 3.84	15.3 ± 0.29	14.3 ± 0.37
Mean		29.5	28.0	18.3	17.6	138.0	130.4	14.0	13.2
75	0	26.5 ± 1.05	25.2 ± 1.00	18.0 ± 1.00	17.1 ± 0.95	131.7 ± 1.01	126.4 ± 1.25	14.4 ± 0.12	13.7 ± 0.11
	50	35.6 ± 1.21	33.8 ± 1.15	20.0 ± 1.00	20.3 ± 0.58	142.6 ± 3.86	135.4 ± 3.66	15.7 ± 0.47	14.9 ± 0.45
	75	37.6 ± 1.72	36.3 ± 1.19	24.7 ± 1.53	23.6 ± 1.21	156.2 ± 6.58	148.4 ± 6.25	17.3 ± 0.46	16.7 ± 0.47
Mean		33.2	31.8	20.9	20.3	14,143.5	136.7	15.8	15.1
TRP	0	25.1	23.9	16.7	15.7	127.6	121.5	12.6	12.0
	50	29.8	28.3	18.3	17.9	137.0	129.9	14.0	13.2
	75	33.3	31.8	21.4	20.7	145.1	138.6	15.0	14.3
L.S.D at 5%	GLY	1.76	0.88	0.64	0.79	5.04	4.65	0.47	0.46
	TRP	1.05	1.34	0.82	0.67	3.90	2.77	0.40	0.52
	Interaction	1.82	2.33	1.41	1.16	6.76	4.80	0.69	0.90

In Table 3, it was shown that the best results for vegetative growth characteristics of celery plant were given by TRP at a concentration of 75 mg/L, followed by a significant difference of 50 mg/L, then 0 mg/L (control).

Observably, the highest values of all measured plant vegetative growth characteristics were recorded by using (GLY + TRP) mix at the highest level of 75 mg/l which was with significant differences from other treatments, while the lowest given data resulted out of the control treatment (Table 3).

### Yield

**Fig. 2:**
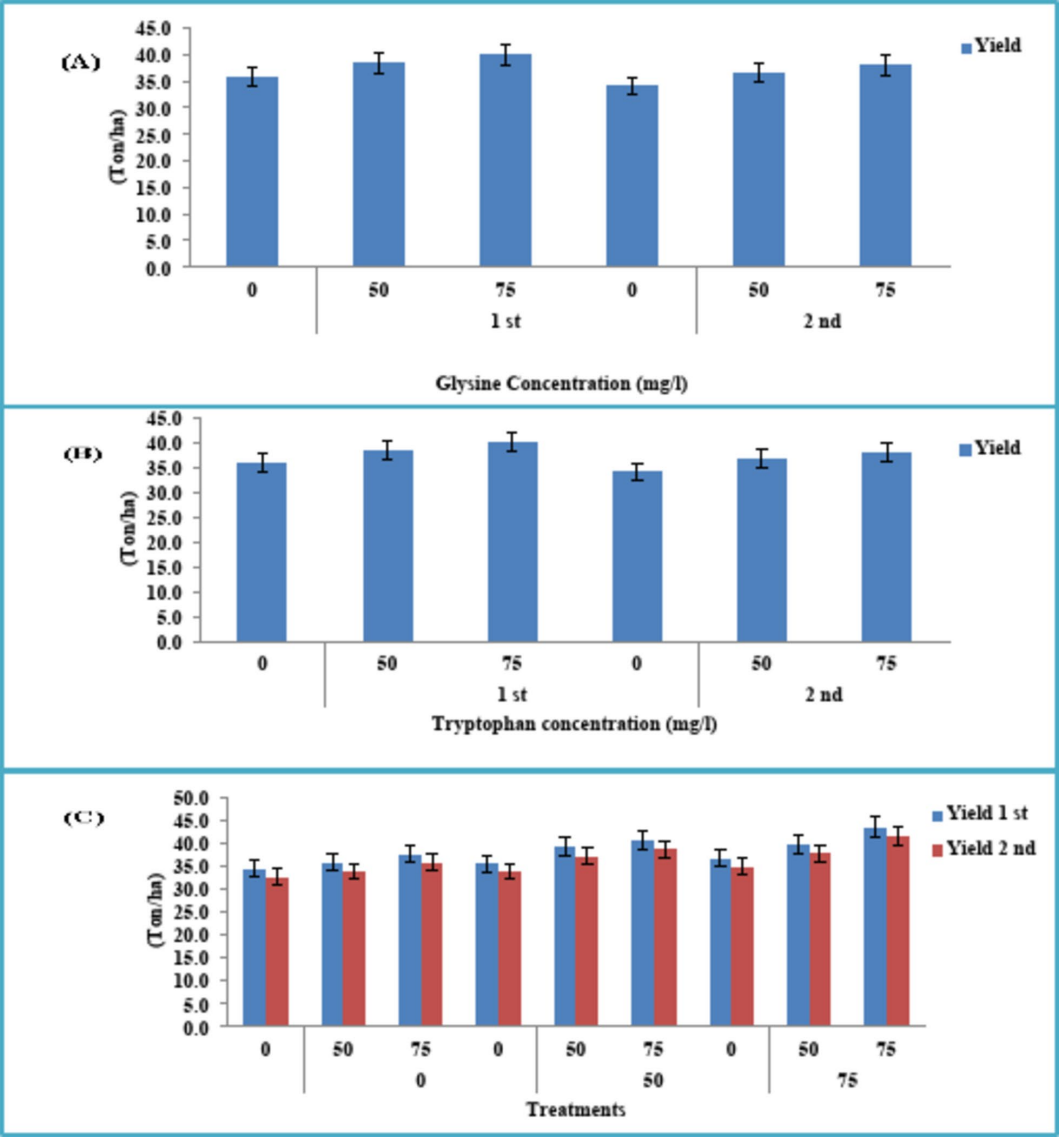
Effect of different concentrations of GLY (**A**), TRP(**B**), and their interaction (**C**) on yield of celery plant during two consecutive seasons 2021/2022–2022/2023.

Figure 2 shows the effect of amino acids as a foliar spray on celery yield per ton/ ha. GLY amino acid enhanced yield per ton/ha (40.0 and 38.0 ton/ ha) using the highest concentration of 75 mg/l, during two seasons 2021/2022 and 2022/2023 (Fig. 2A).

In the same context, TRP registered the maximum productivity (40.6 and 38.6 ton/ ha) as compared to other concentrations, during the two study seasons (Fig. 2 B).

Foliar application of amino acids mixture (GLY + TRP) at a higher concentration of 75 mg/l boosted the productivity of the celery plant (43.6 and 41.4 ton/ ha), during two sequential seasons. In contrast, the lowest yield values were recorded in the control treatment (Fig. 2C).

### Photosynthetic pigments

**Fig. 3:**
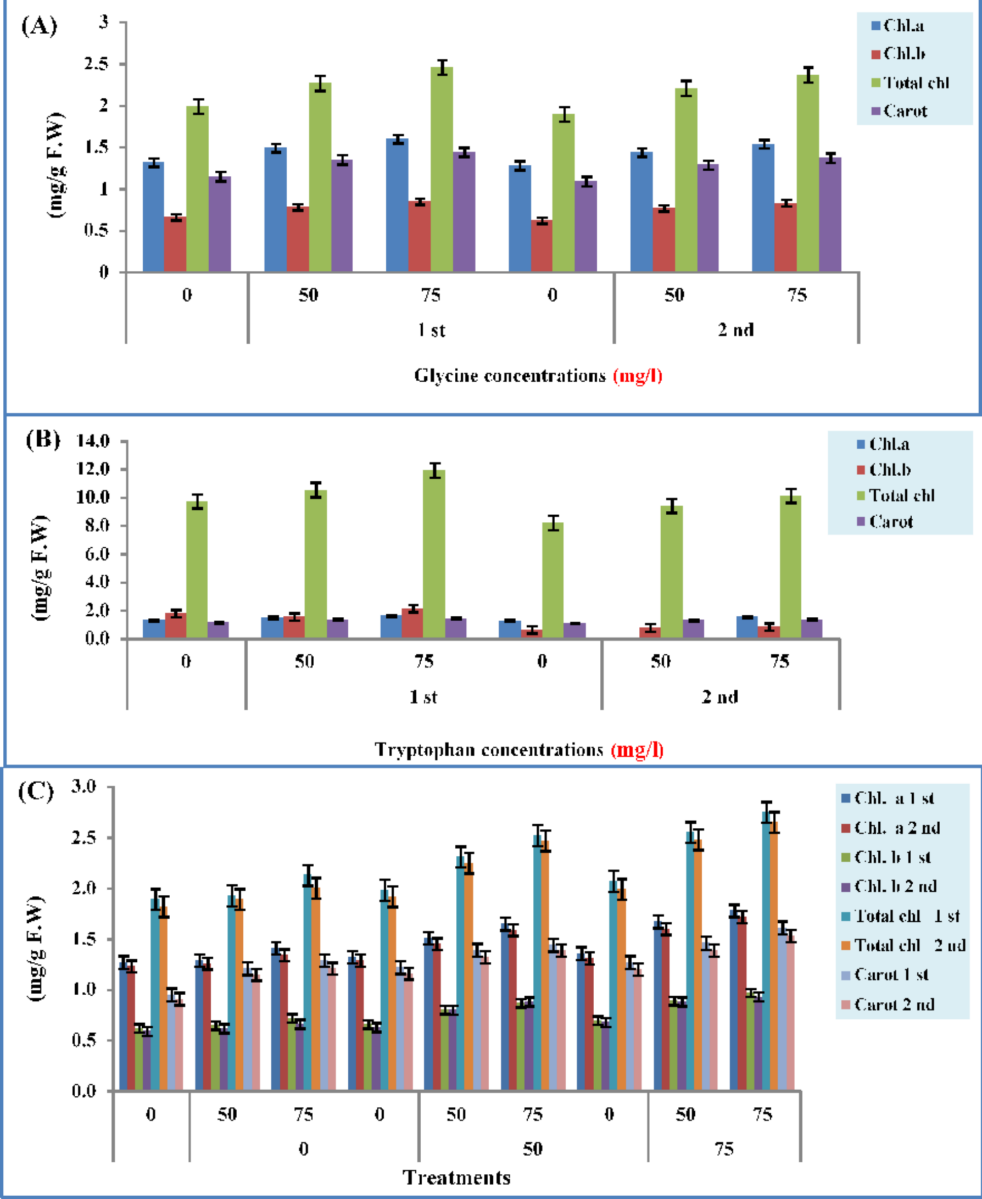
Effect of different concentrations of GLY (**A**), TRP (**B**), and their interaction (**C**) on photosynthetic pigments of celery leaves during two seasons 2021/2022–2022/2023.

Figure 3 presents the amino acids applications impact on the leaf’s photosynthetic pigments (chlorophyll a, b, total chlorophyll, and carotenoids) content, in both study seasons. Chlorophyll a (1.60 and 1.54 mg/g fresh weight), chlorophyll b (0.85 and 0.83 mg/g fresh weight), total chlorophyll (2.46 and 2.37 mg/g fresh weight), and carotenoids (1.44 and 1.37 mg/g fresh weight) showed a valuable increment in its content in plant leaves treated by the GLY amino acid during two growing seasons 2021/2022 and 2022/2023 at the highest level (75 mg/l) these recorded increments were more significant than all other used concentrations (Fig. 3A).

The highest level (75 mg/l) of TRP gave the best values of photosynthetic pigments content as expressed as chlorophyll a (161 and 1.55 mg/g fresh weight), chlorophyll b (0.85 and 0.82 mg/g fresh weight), total chlorophyll (2.47 and 2.37 mg/g fresh weight), and carotenoids (1.45 and 1.38 mg/g fresh weight) followed by the 50 mg/l level and then by 0 mg/l (control), respectively during two experimental seasons, these consequence showed significant (Fig. 3B).

GLY + TRP mix with the highest level (75 mg/l) gave the greatest content of chlorophyll a (1.78 and 1.72 mg/g fresh weight), chlorophyll b (0.97 and 0.93 mg/g fresh weight), total chlorophyll (2.75 and 2.65 mg/g fresh weight), and carotenoids (1.61 and 1.53 mg/g fresh weight) content, during two investigational seasons, as compared to other concentrations (Fig. 3C).

### Phytochemicals content

Table 4; shows that in both seasons (2021–2022) and (2022–2023), the tested biochemical constituents i.e., vitamin C (6.61 and 6.53 mg/100g fresh weight), total phenols (9.79 and 9.30 mg GAE/g dry weight), and total flavonoids (2.85 and 2.74 mg QCE /g dry weight), total antioxidant activity (8.21 and 7.88 µmol Trolox/g dry weight),and total indoles (10.73 and 10.21 mg IAA/g dry weight) contents were enhanced after plants got treated with GLY with the highest level of 75 mg/l in both seasons of this study.

**Table 4:** Effect of GLY and TRPand its different levels on celery’s phytochemicals content during the two seasons of (2021/2022) and (2022/2023).

GLY (mg/l)	TRP (mg/l)	Vitamin C (mg/100g fresh weight)		Total phenols (mg GAE/g dry weight)		Total flavonoids (mg QCE/g dry weight)		Total antioxidant activity (μmol Trolox/ g dry weight)		Total indoles (mg IAA/g dry weight)	
		2021/2022	2022/2023	2021/2022	2022/2023	2021/2022	2022/2023	2021/2022	2022/2023	2021/2022	2022/2023
0	0	4.77 ± 0.21	4.70 ± 0.26	7.13 ± 0.37	6.91 ± 0.33	1.55 ± 0.05	1.52 ± 0.03	5.64 ± 0.43	5.47 ± 0.29	8.88 ± 0.13	8.58 ± 0.37
	50	5.23 ± 0.21	5.00 ± 0.20	8.10 ± 0.10	7.89 ± 0.11	1.72 ± 0.05	1.63 ± 0.04	6.64 ± 0.32	6.36 ± 0.26	9.71 ± 0.22	9.15 ± 0.14
	75	5.90 ± 0.10	5.67 ± 0.15	8.72 ± 0.20	8.28 ± 0.19	1.93 ± 0.06	1.84 ± 0.03	7.43 ± 0.22	7.06 ± 0.21	9.9 ± 0.09	9.45 ± 0.06
Mean		5.30	5.12	7.98	7.69	1.73	1.67	6.57	6.30	9.48	9.06
50	0	4.97 ± 0.13	4.80 ± 0.26	7.72 ± 0.12	7.34 ± 0.11	1.74 ± 0.03	1.67 ± 0.01	6.51 ± 0.25	6.20 ± 0.20	8.84 ± 0.16	8.68 ± 0.28
	50	5.73 ± 0.23	5.57 ± 0.21	9.06 ± 0.05	8.80 ± 0.18	2.53 ± 0.08	2.49 ± 0.04	7.16 ± 0.11	6.81 ± 0.10	10.15 ± 0.26	9.85 ± 0.14
	75	6.50 ± 0.10	6.47 ± 0.25	10.05 ± 0.05	9.55 ± 0.05	3.18 ± 0.02	3.10 ± 0.05	8.37 ± 0.19	7.98 ± 0.18	10.84 ± 0.14	10.56 ± 0.40
Mean		5.73	5.61	8.95	8.56	2.48	2.42	7.35	7.00	9.94	9.70
75	0	5.50 ± 0.10	5.47 ± 0.12	8.99 ± 0.08	8.54 ± 0.07	1.95 ± 0.05	1.86 ± 0.04	7.29 ± 0.18	6.93 ± 0.17	9.84 ± 0.05	9.38 ± 0.11
	50	6.56 ± 0.13	6.50 ± 0.10	9.77 ± 0.11	9.28 ± 0.10	3.15 ± 0.05	3.05 ± 0.05	8.29 ± 0.14	7.99 ± 0.13	10.77 ± 0.10	10.23 ± 0.09
	75	7.73 ± 0.21	7.63 ± 0.15	10.62 ± 0.11	10.09 ± 0.04	3.46 ± 0.06	3.32 ± 0.02	9.03 ± 0.27	8.73 ± 0.35	11.59 ± 0.32	11.01 ± 0.30
Mean		6.61	6.53	9.79	9.30	2.85	2.74	8.21	7.88	10.73	10.21
TRP	0	5.08	4.99	7.95	7.60	1.75	1.68	6.48	6.20	9.18	8.88
	50	5.84	5.69	8.98	8.66	2.47	2.39	7.36	7.05	10.21	9.74
	75	6.71	6.59	9.80	9.31	2.86	2.76	8.28	7.92	10.77	10.34
L.S.D at 5%	GLY	0.18	0.20	0.14	0.20	0.04	0.03	0.48	0.39	0.26	0.30
	TRP	0.15	0.23	0.20	0.18	0.06	0.05	0.21	0.20	0.19	0.26
	Interaction	0.25	0.40	0.35	0.31	0.10	0.08	N.S	N.S	0.33	0.45

N.S = Not Significant (p < 0.05).

The use of TRP with the uppermost concentration of 75 mg/l as foliar implementation recorded a significant effect at LSD 0.05 on the content of vitamin C (6.71 and 6.59 mg/100g fresh weight), total phenols (9.80 and 9.31 mg GAE/g dry weight), and total flavonoids (2.86 and 2.76 mg QCE /g dry weight), total antioxidant activity (8.28 and 7.92 µmol Trolox/g dry weight), and total indoles (10.77 and 10.34 mg IAA/g dry weight) and that was true in the two seasons of this study (Table 4).

Leading by far, using the highest level of 75 mg/l of (GLY + TRP) mix the best values of vitamin C (7.73 and 7.63 mg/100g fresh weight), total phenols (10.62 and 10.09 mg GAE/g dry weight), total flavonoids (3.46 and 3.32 mg QCE /g dry weight), total antioxidant activity (9.03 and 8.73 µmol Trolox/g dry weight), and total indoles (11.59 and 11.01 mg IAA/g dry weight), while the control treatment gave the lowest recorded values (Table 4).On the contrary, there were no significant differences between TRP and GLY regarding its effect on total antioxidant activity contents in both seasons of this study.

### Minerals and protein content

The minerals (nitrogen, phosphorus, potassium) and protein content of celery plants were enhanced by spraying with amino acids as shown in Table 5. The best percentage of nitrogen (2.37 and 2.30%), phosphorus (0.15 and 0.14%), potassium (2.81and 2.71%), and protein (14.60 and 14.17%) was shown with GLY at a concentration of 75 mg/l, respectively during the 2021/2022 and 2022/2023 planting seasons.

**Table 5:** Effect of GLY and TRP and its different levels on celery’s minerals and protein content during the two seasons of (2021–2022) and (2022–2023).

GLY (mg/l)	TRP (mg/l)	N (%)		P (%)		K (%)		Protein (%)	
		2021/2022	2022/2023	2021/2022	2022/2023	2021/2022	2022/2023	2021/2022	2022/2023
0	0	1.27 ± 0.04	1.23 ± 0.02	0.11 ± 0.01	0.10 ± 0.01	1.95 ± 0.05	1.89 ± 0.04	7.79 ± 0.22	7.58 ± 0.14
	50	1.63 ± 0.04	1.55 ± 0.03	0.12 ± 0.02	0.11 ± 0.01	2.30 ± 0.05	2.23 ± 0.04	10.05 ± 0.22	9.55 ± 0.20
	75	1.75 ± 0.05	1.66 ± 0.04	0.13 ± 0.01	0.12 ± 0.01	2.40 ± 0.03	2.31 ± 0.04	10.74 ± 0.28	10.21 ± 0.25
Mean		1.55	1.48	0.12	0.11	2.22	2.15	9.53	9.11
50	0	1.96 ± 0.04	1.91 ± 0.03	0.13 ± 0.01	0.11 ± 0.01	2.26 ± 0.04	2.17 ± 0.02	12.03 ± 0.23	11.77 ± 0.20
	50	2.23 ± 0.08	2.12 ± 0.08	0.14 ± 0.01	0.12 ± 0.01	2.54 ± 0.04	2.41 ± 0.03	13.74 ± 0.33	13.06 ± 0.11
	75	2.35 ± 0.05	2.23 ± 0.05	0.16 ± 0.01	0.14 ± 0.01	2.91 ± 0.02	2.80 ± 0.02	14.45 ± 0.31	13.74 ± 0.28
Mean		2.18	2.09	0.14	0.12	2.57	2.46	13.41	12.85
75	0	2.11 ± 0.04	2.05 ± 0.05	0.13 ± 0.01	0.12 ± 0.02	2.44 ± 0.03	2.34 ± 0.06	12.96 ± 0.25	12.59 ± 0.12
	50	2.33 ± 0.03	2.31 ± 0.03	0.16 ± 0.01	0.14 ± 0.01	2.87 ± 0.03	2.76 ± 0.04	14.35 ± 0.15	14.21 ± 0.16
	75	2.68 ± 0.04	2.55 ± 0.03	0.17 ± 0.02	0.15 ± 0.01	3.12 ± 0.03	3.03 ± 0.06	16.50 ± 0.22	15.71 ± 0.17
Mean		2.37	2.30	0.15	0.14	2.81	2.71	14.60	14.17
TRP	0	1.78	1.73	0.12	0.11	2.22	2.13	10.93	10.64
	50	2.07	2.00	0.14	0.13	2.57	2.47	12.71	12.27
	75	2.26	2.15	0.15	0.14	2.81	2.72	13.90	13.22
L.S.D at 5%	GLY	0.05	0.02	0.01	0.02	0.06	0.06	0.32	0.14
	TRP	0.04	0.03	0.01	0.01	0.03	0.04	0.26	0.14
	Interaction	0.07	0.05	N.S	N.S	0.06	0.07	0.46	0.24

N.S = Not Significant (p < 0.05).

The maximum concentration of TRP 75 mg/l gave an increase in the ratio of nitrogen (2.26 and 2.15%), phosphorus (0.15 and 0.14%), potassium (2.81 and 2.72%), and protein (13.90 and 13.22%), respectively during two trail seasons 2021/2022 and 2022/2023 (Table 5).

The highest percentages of nitrogen (2.68 and 2.55%), phosphorus (0.17 and 0.15%), potassium (3.12 and 3.03%), and protein (16.50 and 15.71%) were recorded with the highest concentration (75 mg/l) of GLY + TRP, during two consecutive growing seasons of 2021 and 2022 as compared to spraying amino acids individually (Table 5). At the same time, nitrogen, potassium, and protein showed significant differences at 5% in the interaction between GLY + TRP, while phosphorus did not show any significant differences in the interaction between GLY + TRP.

### Amino acids content

HPLC analysis indicated the detection of 17 amino acids; Aspartic (ASP), Glutamic (GLU), Serine (SER), Histidine (HIS), Glycine (GLY), Threonine (THR), Arginine (ARG), Alanine (ALA), Threonine (TYR), Cystine (CYC), Valine (VAL), Methionine (METH), Phenylalanine (PHE), Isoleucine (ILE), Leucine (LEU), Lysine (LYS), and Proline (PRO)in the celery plant on a dry basis under different concentrations of GLY and TRP (Table 6). ASP and METH appeared the best value with the highest concentration of TRP (75 mg/l) as compared to GLY. In contrast, the maximum values of GLU, SER, HIS, GLY, THR, ARG, ALA, TYR, VAL, PHE, ILE, LEU, LYS, and PRO showed the interaction between the concentration of GLY up to 75 mg/l and TRP up to 50 mg/l. In contrast, CYC recorded non-detection under various concentrations of GLY and TRP.

**Table 6:** Effect of different concentrations of GLY, TRP, and their interaction on the content of amino acids in the celery plants.

GLY (mg/l)	TRP (mg/l)	Amino acids concentration(ug/g)																
		ASP	GLU	SER	HIS	GLY	THR	ARG	ALA	TYR	CYS	VAL	METH	PHE	ILE	LEU	LYS	PRO
0	0	480.7	ND	35.3	4.5	76.1	31.2	77.0	69.3	65.9	ND	41.4	65.7	184.4	44.7	137.4	191.0	47.7
	50	79.2	55.9	35.4	7.3	59.0	31.8	54.9	47.9	37.1	ND	43.9	60.8	170.6	38.4	139.0	53.1	23.4
	75	555.9	382.1	127.5	18.1	209.6	86.1	149.4	183.4	140.5	ND	171.3	166.8	559.1	138.3	475.5	182.0	208.5
50	0	ND	67.3	38.4	7.9	67.2	25.9	65.0	56.5	46.1	ND	51.5	69.1	181.6	40.2	151.0	53.5	23.5
	50	ND	75.9	24.6	4.0	41.4	19.5	46.5	36.1	29.9	ND	41.6	45.2	126.6	28.7	98.0	36.0	15.2
	75	ND	55.1	28.8	5.8	51.5	25.9	50.1	43.8	31.4	ND	45.1	52.9	136.4	31.5	109.4	26.2	21.4
75	0	ND	50.9	63.7	11.1	109.0	57.2	115.0	94.2	70.5	ND	77.2	98.1	270.3	67.2	237.1	71.1	36.0
	50	355.0	551.2	205.7	190.1	581.0	285.9	361.9	267.2	189.6	ND	445.9	123.5	614.2	238.4	581.6	316.9	6420.5
	75	227.9	361.1	162.9	145.8	461.3	252.2	291.6	216.8	174.3	ND	360.6	119.9	538.8	182.7	445.1	233.9	6290.9

ND = Not detected.

## Discussion

Our present study was established to investigate the amino acids (GLY and TRP) effect on celery plant growth, yield, and quality under Egyptian conditions. The recorded results regarding plant length, number of leaves per plant, fresh weight of leaves g per plant and dry matter showed an impressive positive enhancement on all evaluated growth characteristics with the amino acids GLY at concentration of 75 mg/l. These consequences can be attributed to the GLY improves photosynthesis efficiency with the same concentration in this study by enhancing chlorophyll formation and encouraging plant’s vegetative growth^42^**.**Similarly, Rosa et al.^13^ found that L-glycine can affect butter head lettuce growth and yield as the highest head weight, root weight, and number and length of leaves values resulted from plants sprayed with 80 to 120 mg/l foliar doses. In cucumber, GLY improved leaves characteristics such as water content and photosynthesis, its effect on optimizing leaves photosynthesis efficiency might be the reason for gaining higher cucumber yield^43,44^**.**Thus increasing the celery yield per hectare at the concentration of 75 mg/l. Shooshtari et al.^24^ reported that either soil or foliar GLY applications were able to improve cucumber yield in limed soil conditions. Furthermore, GLY boosted the content of vitamin C, total phenols, total flavonoids, total antioxidant activity, total indoles, minerals, protein, and amino acids at a concentration of 75mg/l may be due to GLY has many mechanisms that might enhance plant growth, and nutrient uptake besides photosynthetic activity which is associated with bio-stimulation, also it has signaled and protective effects on plants^43^. As well as having semi-hormonal effects on plant growth^45^.

In the same vein, the amino acid TRP improved the vegetative growth characteristics studied in celery plants with the highest concentration of 75mg/l may be due to enhanced photosynthesis pigments with the maximum concentration (75 mg/l). Therefore, the celery yield per hectare increased as a result of increased vegetative growth and chlorophyll for the same treatment. Baqir et al.^42^ mentioned the importance of TRP helping indole acetic acid formation which is necessary for growth and plays a role in the early yield. Also, that was found by Gondek and Mierzwa-Hersztek^46^who said that TRP promotes plant growth even by using low concentration, due to its action as a precursor indole acetic acid growth hormone. Mustafa et al.^47^ stated that applying TRP either in soil or to leaves as foliar application resulted in a significant increase in plant height by 16.9 percent in foliar application and by 58.4% in soil application. The same effect of TRP was mentioned by Ibraheim and Mohsen^48^stated that lettuce foliar spraying by TRP at rates of 30 or 45 mg/l had a significant increment on plant’s height, leaves number per plant, fresh weight, leaf area, dry weight and chlorophyll content. Those results might relate to the physiological role that TRP plays in plant growth by stimulating cell divisions in the apical meristem and its circuitous role in influencing the natural synthesis of plant auxin. Furthermore, Sarah et al.^49^found foliar application of TRP at 75 mg/l in concentration increased pod number/plant by 4.3%, and seeds/pod by 12.9% for broad beans. Similarly, Zhong et al.^50^ reported that spraying strawberries with TRP increased yield, and enhanced fruit quality, also, Frankenberger et al.^51^ found that TRP increases radish yield significantly by 15% and 18% when it was treated by 20.4 and 204 mg/m^-2^, respectively. According to Ahmed et al.^52^, the increase in the shoot dry matter after TRP treatment might be because of increased cell expansion, leaf area and leaves number per plant. As for the effect of TRP on the content of biochemical constituents in the celery plant, TRP augmented the value of vitamin C, total phenols, total flavonoids, total antioxidant activity, total indoles, minerals, protein, and ASP, and METH with the highest concentration of 75 mg/l. These results might be due to use TRP increases many hormone levels in tissues, i.e., of IAA, GA, and ABA^18^. Moreover, Khattab et al.^23^ mentioned that TRP helps IAA formation and has a valuable function in early growth. Meanwhile, the protein content increase in crops in response to TRP foliar application was mentioned by Dawood and Sadak^22^in rapeseed plants(by 8.2%)when used 75 mg/l TRP, and in wheat(by18.5%) by using 50 mg/l TRP^53^.

Respecting the interaction between amino acids treatments in the current study showed a superior influence on celery’s plant growth, yield, and biochemical constituents, it has been clear that (GLY + TRP) mixture on the highest application level of 75 mg/l raised positively with significant values celery’s growth, and yield, in both seasons of the study that might refer to its effect influence plant growth, nutrients absorption, and uptake, photosynthesis efficiency as well as the hole plants nutritional status, Increases deferent crops biomass and yield by foliar amino acids treatments have also been mentioned by Souri et al.^45^that were consistent with Rosa et al**.**^9^who found that the use of foliar amino acid can encourage the absorption of many nutrients which in turn affects the yield. Similar results were reported by Khan et al.^54^; and Noroozlo et al.^27,29^ who noticed higher values of total yield. Lettuce growth improved by applying GLY and/or other amino acids enhanced plant yield in different studies by Khan et al.^54^ While Mustafa et al.^47^ stated that applying TRP either in soil or to leaves as foliar application increased the fruit yield of okra by 17% in soil application and by 95.5% in foliar application. This result may also be attributed to the fact that the high value of leaves’ chlorophyll content is probably due to amino acids tonic effect on chlorophyll biosynthesis and simultaneously decreases its degradation^45^. Abd El-Rheem^55^ stated that amino acid foliar spraying at 250–500 mg/l concentrations led to raised chlorophyll a, b in lettuce leaves. On the positive side effect of (GLY + TRP) mixture at the highest tested level of 75 mg/l used on the biochemical constituents may be related to the enhancement that happened to the celery plant’s nutritional status as a result of the effect of these amino acids on the plant’s chlorophyll content and plant hormones. This is reflected as an increase of vitamin C, total phenols, total flavonoids, total antioxidant activity, total indoles, minerals, protein, and amino acids content. Francke et al.^56^, reported that amino acids have several effects on vitamin C content, relying on crop species and its edible parts. At the same time, Foliar applications of GLY and glutamine, at levels of 250 and 500 mg/l, had a significant increase in cucumber leaves vitamin C contents^24^.In the same direction, the content of phenolic acids and flavonoids is linked to antioxidant activity^57^. Taraseviciene et al.^58^stated that relying on mint variety, all tested amino acids treatments increased phenolic compound values compared with control. The efficiency of TRP 100 mg/l in M. piperita “Swiss” was 3.51 as TRP is a precursor for alkaloids, glucosinolates, phytoalexins as well as auxins. In coriander, different GLYconcentrations of 5, 10, 20, and40 mg/l had a significant influence on increasing leaf antioxidant activities^28^. In the context, amino acids boost plant tissues protein concentration because of nitrogen involvement in protein structure and nucleic acid synthesis. Also, Hafez et al.^59^stated that amino acids supply plant cells with absorbable nitrogen, which encourages protein synthesis. Also, amino acid increased the protein content in Fabaceae plants bean seeds^60^. From a nutritional point of view, higher leaf content of protein is desirable regarding metabolic reaction and optimum photosynthesis activity^25^.Nevertheless, it is clear that either foliar or soil treatments of GLY increased leaf protein and that might happen through enhancements of soil minerals availability, uptakes, translocations, and distributions in plant tissues^43^.

We conclude from the previous study that antioxidants represented by vitamin C, phenols, flavonoids, and the total content of antioxidant activity were analyzed to estimate these components present in celery plants because it is a plant characterized by a high content of antioxidants, as well as to study the extent of the effect of these amino acids on the plant’s content of them. In the same context, the total content of indoles was estimated due to the importance of the amino acid tryptophan in building plant hormones (Indole acetic acid) , which increases with improved plant growth^57^. As for the nutrients and amino acids, it was found that they are affected by spraying plants with amino acids.

## Conclusion

It can be surely reported that amino acids (GLY and TRP) have an encouraging influence on the celery plant’s growth, yield, nutritional status, and quality. That effect was clear when both were used as a mix instead of individual use, and that was at the highest tested level of foliar application. Spraying (GLY + TRP) amino acids mix at 75mg/l of concentration led to promote all studied vegetative growth characteristics, yield, photosynthetic pigments (chlorophyll a, b, total chlorophyll and carotenoids) contents, biochemical constituents (vitamin C, total phenols, total flavonoids, total antioxidant activity, total indoles, minerals, protein, and amino acids) content of celery plant. Amino acids use could be the best possible choice to improve the production of celery to its best performance.

## Data Availability

The original contributions presented in the study are included in the article, further inquiries can be directed to the corresponding author. The datasets used and/or analyzed during the current study are available from the corresponding author on reasonable request.
